# Descending Colon Perforation Due to Ingestion of Foreign Body

**DOI:** 10.7759/cureus.47479

**Published:** 2023-10-22

**Authors:** Christos Tepelidis, Panagiotis Fotiadis, Athanasios Permekerlis, Trigona Karastergiou, Petros Kouridakis

**Affiliations:** 1 2nd Surgical Department, 424 General Military Hospital, Thessaloniki, GRC; 2 Surgery, General Hospital of Thessaloniki "George Papanikolaou", Thessaloniki, GRC

**Keywords:** descending colon, laparoscopic treatment, acute peritonitis, swallowed foreign body, intestinal perforation

## Abstract

Foreign body ingestion is a common reason for emergency department (ED) visits, with rare complications necessitating immediate surgical intervention. This case report discusses diagnosis and treatment, emphasizing the importance of prompt intervention. A 45-year-old male with dentures presented with acute left abdominal pain. Diagnostic tests identified a foreign body in the descending colon, leading to laparoscopic surgery. Early laparoscopy offers a safe and reliable alternative to exploratory laparotomy. This case underscores the significance of swift diagnosis, preventing severe complications like peritonitis, obstruction, and hemorrhage. In conclusion, while foreign body ingestion is common, intestinal perforation remains extremely rare. Physicians should consider it in their differential diagnosis, with computed tomography (CT) and rapid surgical intervention as crucial components of proper management.

## Introduction

Foreign body ingestion is a common clinical occurrence affecting diverse demographic groups, presenting unique challenges for healthcare practitioners. This phenomenon imposes a significant burden on global healthcare systems. Nearly 80% of individuals who ingest foreign objects pass them through the gastrointestinal tract without complications [1,2], while approximately 20% may encounter issues such as obstruction, perforation, or hemorrhage, necessitating medical intervention [3]. Gastrointestinal perforation, although exceedingly rare (occurring in fewer than 1% of cases), carries substantial severity [4,5]. In the United States, an estimated 1500 individuals annually suffer the consequences of foreign body ingestion, emphasizing the critical need for understanding this clinical phenomenon [6].

Specific demographic groups, including children, adolescents, the elderly, individuals with mental health issues, and those with oral or dental conditions, exhibit a higher prevalence of foreign body ingestion [4,6]. Denture wearers, individuals dealing with alcoholism or drug addiction, and those who rapidly consume food are particularly susceptible [5,7].

Assessing the attributes of the ingested foreign body is crucial when considering the likelihood of complications, especially perforation. Risk significantly escalates when the foreign object is elongated and possesses sharp characteristics, such as fish bones, chicken bones, or toothpicks [6,8,9]. Perforations predominantly manifest in areas marked by natural bends, constrictions, and specific locations like the ileocecal valve. Additionally, the sigmoid colon, while part of the large intestine, is also susceptible to perforation due to foreign body ingestion [10]. The size, shape, and composition of the foreign object are critical determinants of complication severity.

In instances where complications, such as perforation, arise due to foreign body ingestion, surgical intervention becomes crucial. Surgical procedures are typically deemed necessary when endoscopic retrieval proves ineffective or when complications mandate immediate surgical attention [5]. Minimally-invasive surgical techniques, notably laparoscopy, have shown promise in managing complex foreign body ingestion cases, offering advantages such as reduced post-operative recovery periods and improved patient outcomes [11].

Subsequent sections of this case report will delve into a specific clinical presentation, highlighting the challenges and management of foreign body ingestion, with a focus on the significance of timely diagnosis and appropriate intervention to mitigate potentially life-threatening complications.

This study was previously presented as an ePoster at the 31st International European Association of Endoscopic Surgery (EAES) Congress, contributing to the dissemination of knowledge in the field of gastroenterology and minimally invasive surgery.

## Case presentation

A 45-year-old patient presented to the emergency department (ED) with abdominal pain localized in the left lower quadrant. The pain had commenced an hour prior, and no other accompanying symptoms such as nausea, changes in bowel habits, or fever were reported. During the medical history interview, the patient mentioned that he had accidentally swallowed a chicken bone approximately three hours before his visit. Notably, the patient wore dental prostheses, and there was no history of any other medical conditions or surgeries.

**Figure 1:**
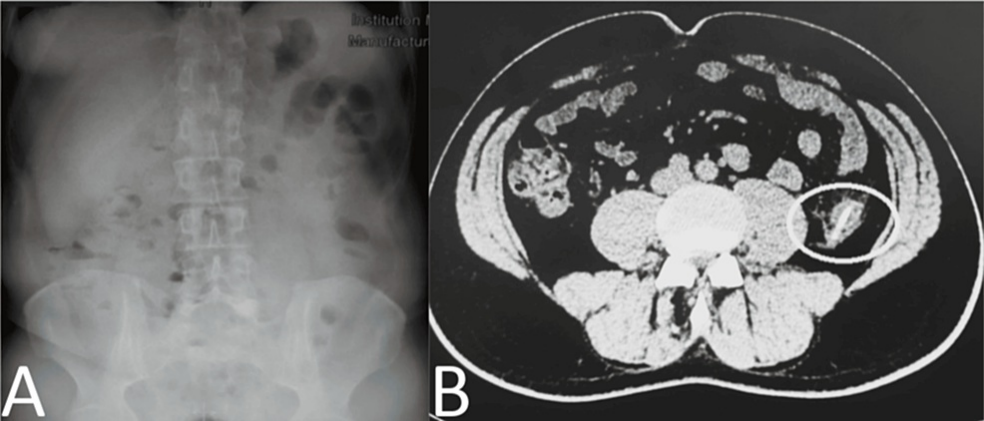
Abdominal X-ray and CT of the patient. (A) Abdominal X-ray revealed no abnormal findings, including the absence of free intra-peritoneal air. (B) CT imaging findings of foreign body penetration into the descending colon.

The patient appeared well-nourished and in good general condition. His vital signs were as follows: BP was 140/75 mmHg, HR was 80 beats/min, and he was afebrile (temperature: 36.8°C). Clinical examination revealed tenderness on deep palpation of the left lower abdominal quadrant and the corresponding inguinal fossa. Additionally, rebound tenderness was noted, but there were no signs of involuntary muscle contractions or peritoneal irritation. Bowel sounds were within the normal range. Subsequent laboratory findings indicated hematocrit (Hct) of 48% (normal range: 38-52%), hemoglobin (Hb) level of 17.1 g/dL (normal range: 13.8-17.2 g/dL), white blood cell (WBC) count: 14.40 K/μL (normal range: 4.5-11 k/μL), with neutrophils (NEUT) accounting for 73.9%, C-reactive protein (CRP): 3.87 mg/dL (with a normal reference range below 0.5 mg/dL). Plain film radiograph examinations of the chest and abdomen in an upright position did not reveal any free subdiaphragmatic air or other pathological findings (Figure 1A). Therefore, an abdominal computed tomography (CT) scan with intra-venous contrast was performed, which revealed the following: a foreign body with bone-like density, approximately 4.5 cm in length and 0.5 cm in thickness, had completely penetrated the wall of the colon at the level of the L3-L4 vertebrae. This was accompanied by pericolic fat stranding and the presence of microbubbles, possibly indicative of free intra-abdominal air, although no signs of pneumoperitoneum were observed (Figure 1B).

**Figure 2:**
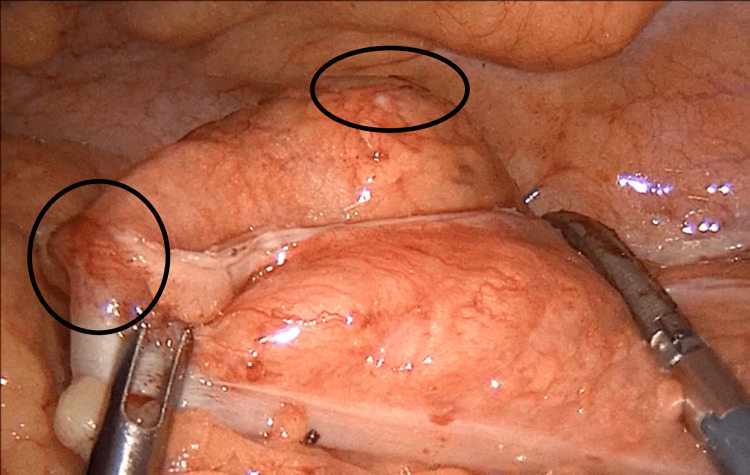
Intra-operative image depicting the affected segment. The recognition of this was aided by the sensation of hardness present on the atraumatic laparoscopic grasper.

**Figure 3:**
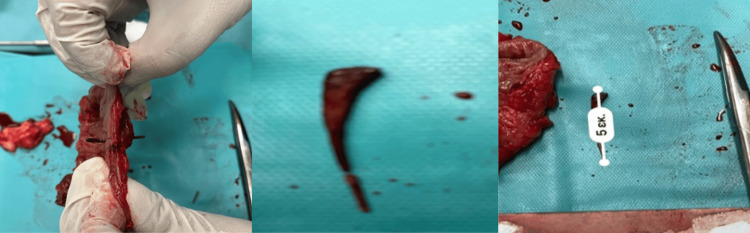
Surgical specimens highlighting the affected segment and the foreign body (chicken bone).

**Figure 4:**
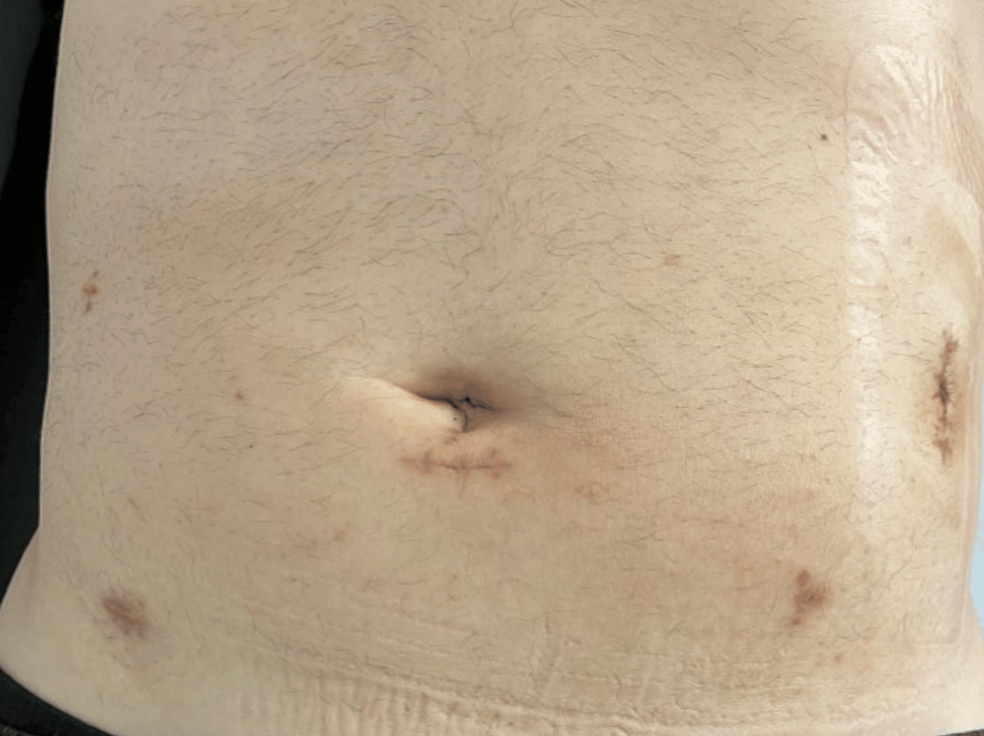
Post-operative image of the patient's abdomen, showing incisions and trocar placement.

Subsequently, the patient underwent intra-venous fluid resuscitation, received antibiotic therapy (piperacillin/tazobactam), and, with his informed consent, was taken to surgery. Initially, a laparoscopic exploration was performed, during which the perforated colonic segment was identified as well as the chicken bone causing the perforation (Figures 2, 3). The peritoneal cavity was meticulously examined and found to be clean, devoid of any signs of purulent fluids, fluid collections, or intestinal distensions. It was decided to proceed with laparoscopic colectomy involving the affected segment and extra-corporeal side-to-side anastomosis, utilizing a wound protector through a paramedian incision. Notably, the sigmoid colon was long enough, obviating the need for mobilization of the splenic flexure to perform the anastomosis. Intra-peritoneal drainage was also placed near the anastomosis site, with its endpoint in the Douglas space. The patient experienced an uneventful recovery and was discharged on the 6th day after surgery (Figure 4). Nonetheless, on the 4th day following the operation, a wound infection manifested at the site of the paramedian incision, necessitating treatment involving surgical debridement and thorough wound care.

## Discussion

The ingestion of a foreign body is a common cause of patient admission to a hospital's ED. It has been observed that there are high-risk groups, such as young children, the elderly, psychiatric patients, alcoholics, and drug addicts, as well as certain predisposing factors [4]. Individuals who eat rapidly and those who wear dental appliances and dentures, in the latter case, experience reduced sensitivity of the oral cavity [12].

The most common foreign bodies described are fish and chicken bones, as well as toothpicks [9]. However, this can vary depending on the dietary habits of each population. The likelihood of complications, including perforation in this case, depends on how elongated, thin, and sharp the object is [8].

The majority of patients with this condition, approximately 80-90%, will be asymptomatic and may require monitoring for a week or endoscopic removal of the object without any complications [5]. A total of 20% of cases may experience some complications, such as obstruction, perforation, bleeding, or the presence of intestinal fistula [12]. Perforation, in particular, accounts for less than 1% of complications from foreign body ingestion and can have very unfavorable outcomes if left untreated [4]. It can lead to localized, regional, and generalized peritonitis, intra-abdominal abscesses, and sepsis.

The parts of the gastrointestinal tract that are most prone to perforation from a foreign body are those with natural angulations, narrower passages, and tight corners [10]. For this reason, the upper and lower esophageal sphincters, the pylorus, the duodenum, the ileocecal valve, the appendix, and the sigmoid colon, are anatomical regions at increased risk of perforation. Additionally, a history of intra-abdominal surgeries (anastomoses, adhesions), the presence of diverticula, and intra-abdominal masses increase the likelihood of complications [7].

The time period between the ingestion of a foreign body and the appearance of symptoms is usually long-term (from several days, in the majority of patients, to months or even years) [13]. However, it has been observed that the more immediate the onset of symptoms, the more acute and severe the patient's clinical condition is [14].

The clinical symptoms of patients with gastrointestinal perforation are typically those of acute abdomen and typically consist of abdominal pain (localized or diffuse), nausea, vomiting, and fever [15]. Laboratory findings indicate leukocytosis and an increase in C-reactive protein (CRP). Plain abdominal X-rays in an upright position rarely reveal the presence of a foreign body, less than 32%, because these objects are small and have limited radiopacity [14,16]. Contrast-enhanced CT imaging seems to be the preferred imaging examination, as it allows for the identification of the site of perforation with approximately 86% sensitivity and the detection of foreign bodies [17]. It practically assists the surgeon in creating a surgical plan. It is worth noting that pneumoperitoneum is rarely present because the perforation tends to be covered by fibrin, omentum, and adjacent loops of the small intestine, leading to containment [13]. The use of oral contrast may hinder the recognition of radiopaque material - the foreign body [18]. The radiological findings of CT usually include a thickened wall, increased mesenteric fat density, and air in the perforation area, usually localized [4]. The differential diagnosis of perforation is similar to that of acute abdomen and depends on the anatomical location of the symptomatology - clinical signs. Typically included are acute appendicitis, acute diverticulitis, hollow viscera perforation, etc.

Antibiotic therapy should be broad-spectrum, and the selection is multifactorial. It depends on the location and timing of the perforation, the degree of contamination of the peritoneal cavity, the patient's age, comorbidities, general condition, and the surgeon's experience [13].

The indications for surgical management of foreign body ingestion are as follows: (1) failure of endoscopic removal, (2) patient unfit for endoscopy, (3) the presence of complications (perforation, bleeding, intra-peritoneal syringe, abscesses, etc.) [19]. In the case of perforation, the choice of surgical technique to be used depends on the affected area. In the case of stomach injuries, primary closure was performed. For injuries to the small intestine, treatment options included primary closure, segmental resection, and anastomosis. Similarly, for injuries affecting the large intestine, the range of treatment modalities comprised primary closure, wound eversion with colostomy, segmental resection, and anastomosis, as well as the consideration of Hartmann's reversal [4,5]. Laparoscopic repair appears to be feasible and safe and has been utilized in numerous cases. Furthermore, it offers the advantage of providing clear visualization and adequate control of the peritoneal cavity, enlargement of the abdominal organs, and easier identification of the injury or foreign body. Additionally, it provides all the well-known advantages of laparoscopic techniques, such as lower rates of post-operative ileus and pain, smaller incisions and superior cosmetic results, shorter hospital stays, faster return to work, and less frequent post-operative hernias [14]. However, it should be noted that laparoscopy has its limitations, such as a history of abdominal surgeries (increased risk of adhesions), technical difficulties with the risk of intra-abdominal organ injury, and the patient's unsuitability (comorbidities) for laparoscopy.

It's essential to highlight that the outcomes of patients with gastrointestinal perforation following foreign body ingestion can vary significantly depending on various factors, including the location and size of the perforation, the type of foreign body, the patient's overall health, and the timeliness of intervention. Studies have reported mortality rates as high as 6.5% and morbidity rates of approximately 24.2% in these cases [18]. However, it's crucial to emphasize that these statistics are approximate and can vary widely based on individual patient factors and the promptness and effectiveness of medical and surgical interventions. Timely diagnosis, appropriate treatment, and a multidisciplinary approach involving gastroenterologists, surgeons, and critical care specialists play a vital role in improving patient outcomes.

## Conclusions

Foreign body ingestion presents a complex clinical scenario, where the risk of perforation looms large. Denture wearers, among other predisposing factors, emphasize the necessity for a meticulous medical history assessment. The advent of laparoscopy has revolutionized both diagnosis and treatment, offering precision and minimal invasiveness. Notably, CT imaging has emerged as a pivotal diagnostic tool, enabling timely interventions. While mortality and morbidity rates may fluctuate, underscoring the importance of early detection, vigilant monitoring, and the evolving landscape of laparoscopic techniques, alongside the indispensable role of CT scans, remain paramount. As medical science advances, adopting a comprehensive and adaptable approach is imperative for effectively addressing this common issue.
